# Sex Differences in Outcome of Trauma Patients Presented with Severe Traumatic Brain Injury: A Multicenter Cohort Study

**DOI:** 10.3390/jcm12216892

**Published:** 2023-11-01

**Authors:** Elise Beijer, Stefan F. van Wonderen, Wietse P. Zuidema, Marieke C. Visser, Michael J. R. Edwards, Michael H. J. Verhofstad, Tjarda N. Tromp, Charissa E. van den Brom, Esther M. M. van Lieshout, Frank W. Bloemers, Leo M. G. Geeraedts

**Affiliations:** 1 Department of Trauma Surgery, Amsterdam UMC, Vrije Universiteit Amsterdam, 1105 AZ Amsterdam, The Netherlands; 2 Department of Anesthesiology, Amsterdam UMC, Vrije Universiteit Amsterdam, 1105 AZ Amsterdam, The Netherlands; 3 Laboratory of Experimental Intensive Care and Anesthesiology, Amsterdam UMC, University of Amsterdam, 1105 AZ Amsterdam, The Netherlands; 4 Department of Intensive Care Medicine, Amsterdam UMC, University of Amsterdam, 1105 AZ Amsterdam, The Netherlands; 5 Department of Neurology, Amsterdam UMC, Vrije Universiteit Amsterdam, 1081 HV Amsterdam, The Netherlands; 6 Department of Trauma Surgery, Radboud University Medical Center, 6500 HB Nijmegen, The Netherlands; 7 Trauma Research Unit, Department of Surgery, Erasmus MC, University Medical Center Rotterdam, 3000 CA Rotterdam, The Netherlands

**Keywords:** sex dimorphism, TBI, mortality, ICU, mechanical ventilation

## Abstract

The objective of this study was to determine whether there is an association between sex and outcome in trauma patients presented with severe traumatic brain injury (TBI). A retrospective multicenter study was performed in trauma patients aged ≥ 16 years who presented with severe TBI (Head Abbreviated Injury Scale (AIS) ≥ 4) over a 4-year-period. Subgroup analyses were performed for ages 16–44 and ≥45 years. Also, patients with isolated severe TBI (other AIS ≤ 2) were assessed, likewise, with subgroup analysis for age. Sex differences in mortality, Glasgow Outcome Score (GOS), ICU admission/length of stay (LOS), hospital LOS, and mechanical ventilation (MV) were examined. A total of 1566 severe TBI patients were included (831 patients with isolated TBI). Crude analysis shows an association between female sex and lower ICU admission rates, shorter ICU/hospital LOS, and less frequent and shorter MV in severe TBI patients ≥ 45 years. After adjusting, female sex appears to be associated with shorter ICU/hospital LOS. Sex differences in mortality and GOS were not found. In conclusion, this study found sex differences in patient outcomes following severe TBI, potentially favoring (older) females, which appear to indicate shorter ICU/hospital LOS (adjusted analysis). Large prospective studies are warranted to help unravel sex differences in outcomes after severe TBI.

## 1. Introduction

Traumatic brain injury (TBI) is a major cause of mortality and morbidity, resulting in millions of years of life lost [1,2,3,4]. In a European cross-sectional study with nearly 1.5 million patients, 55% of the patients suffering from TBI were 44 years or younger. TBI contributed to about 37% of the overall all-injury age-adjusted mortality, and interestingly, when splitting for sex, 42% in males and 29% in females [3]. Male patients were also more frequently admitted to the Emergency Department (ED) after TBI when compared to female patients and comprised the majority of mortalities [3].

In a large retrospective analysis, it was shown that female sex was independently associated with reduced mortality and decreased complications after TBI when compared to their male counterparts, especially in the peri- and postmenopausal-aged [5]. Likewise, female patients aged ≥ 50 years with moderate-to-severe TBI had increased survival rates when compared to age-matched males [6]. Furthermore, it was retrospectively found that female TBI patients had lower mortality rates compared to males during hospital admission [7]. On the other hand, several studies found higher mortality rates for female sex compared to male sex [8,9]. Ottochian et al. showed that females, particularly those aged ≥ 55 years, were significantly associated with mortality in isolated TBI, and another study found that females < 50 years had significantly higher mortality rates than males [8,9]. 

Although clinical studies have shown conflicting results regarding the protective effect of female sex in TBI [5,6,7,8,9,10,11,12,13], there is evidence suggesting that male and female nervous systems respond differently to injury. For example, in preclinical studies, the administration of estrogen and progesterone had a protective effect on mortality and complications, such as edema formation in rodents with TBI [14,15,16,17,18]. Furthermore, previous research found several protective effects of estrogen administration in mice with spinal cord injury, such as reducing the development of inflammation and tissue injury [19]. These findings suggest a role for sex hormones, and consequently age, in the relationship between sex and outcome after TBI, which, together, could possibly explain the contradictory findings of the clinical studies.

The aim of the present study was to investigate whether there is an association between sex and outcome among trauma patients presented with severe TBI. It was hypothesized that female sex, especially in the premenopausal age (aged 16–44 years) subgroup, is associated with better outcomes when compared to their male counterparts.

## 2. Materials and Methods

### 2.1. Study Design and Population 

A retrospective study in three level I trauma centers in the Netherlands between 1 January 2015 and 31 December 2018 was performed. The included patients were aged 16 years or older and were admitted to the ED with a Head Abbreviated Injury Score (AIS) ≥ 4 [20]. Patients with drowning, asphyxia, or burns were excluded. To consider the potential influence of hormonal status, patients were stratified into two age groups (16–44 and ≥45 years) as a surrogate for the premenopausal and perimenopausal/postmenopausal phase, respectively [21]. In addition, a sub-analysis was performed in patients with isolated severe TBI. Isolated severe TBI was defined as an AIS Head ≥ 4 and other body region AIS scores ≤ 2. The study was approved by the independent Medical Research Ethics Committees of the three participating hospitals.

### 2.2. Data Collection and Parameter Outcome 

Demographic and outcome parameters for all male and female patients were collected from the Trauma Registry. Injuries were classified by AIS per body region, injury severity by the Injury Severity Score (ISS), and vital signs by the Revised Trauma Score (RTS). Severe injuries of the head, thorax, abdomen, and lower extremity were dichotomized as an AIS of ≥3 per body region, respectively. ISS was used as a continuous variable. Vital signs of the patients were represented by the RTS as continuous and dichotomous variables (<4). The mechanism of injury (MOI) was either blunt or penetrating. The Glasgow coma scale (GCS) at admission was collected both as continuous and as dichotomous variables (≤8). Systolic blood pressure (SBP) at admission was collected both as a continuous variable and a dichotomized variable (<90 mmHg). Furthermore, pre-trauma comorbidity was collected as an ordinal variable using the American Society of Anesthesiologists (ASA) Physical Status Classification System (healthy, ASA I or mild comorbidity, ASA II and severe comorbidity, ASA ≥ III). Prehospital intubation, Physician-staffed Helicopter Emergency Medical Services (P-HEMS), assistance, and in-hospital treatment (including craniotomy and Intra Cranial Pressure (ICP) measurement) were defined as dichotomous variables. Primary outcomes were in-hospital mortality, unfavorable Glasgow Outcome Score (GOS) ≤3 at hospital discharge [22] and ICU admission. Secondary outcomes were 30-day mortality, ICU LOS, hospital LOS, MV during admission, and MV duration. 

### 2.3. Statistical Analyses

Statistical tests were performed using IBM SPSS Statistics 27.0 and two-sided testing with a *p*-value < 0.0167 and <0.05 for primary and secondary outcomes, respectively, was considered statistically significant. A univariate data analysis was performed to compare patient demographics. In this study, male patients were used as a reference group compared to female patients. Continuous variables were presented as mean ± standard deviation (SD) and independent student’s *t*-tests were used to compare means between two groups for equally distributed data. For skewed data, median and interquartile range (IQR) were shown and a Mann–Whitney U-test was performed to compare medians between two groups. Categorical variables were expressed as proportions and compared using Chi-squared (χ^2^) tests or Fisher’s Exact Test. Logistic regression analyses were performed to determine if sex was associated with in-hospital mortality, GOS ≤ 3, ICU admission, 30-day mortality, ICU LOS > 7 days, hospital LOS > 7 days, MV during admission, and MV > 7 days. Regression models were presented with unadjusted values, and adjusted values for a priori-based covariates based on clinical relevance, including age, MOI, ISS, RTS < 4, GCS ≤ 8, Shock Index (SI), comorbidity ASA ≥ III, ICP measurement, and craniotomy. A maximum number of covariates was accepted during analysis powered to the smallest group in our primary outcome. Missing data was not replaced and only complete cases were included in the multivariable models. Logistic regression analyses results are presented as odds ratio (OR) with 95% confidence intervals (CI). Also, analyses were performed after stratification for age (16–44 and ≥ 45 years) and isolated TBI. 

## 3. Results

### 3.1. Patient Characteristics—Severe TBI

**Figure 1:**
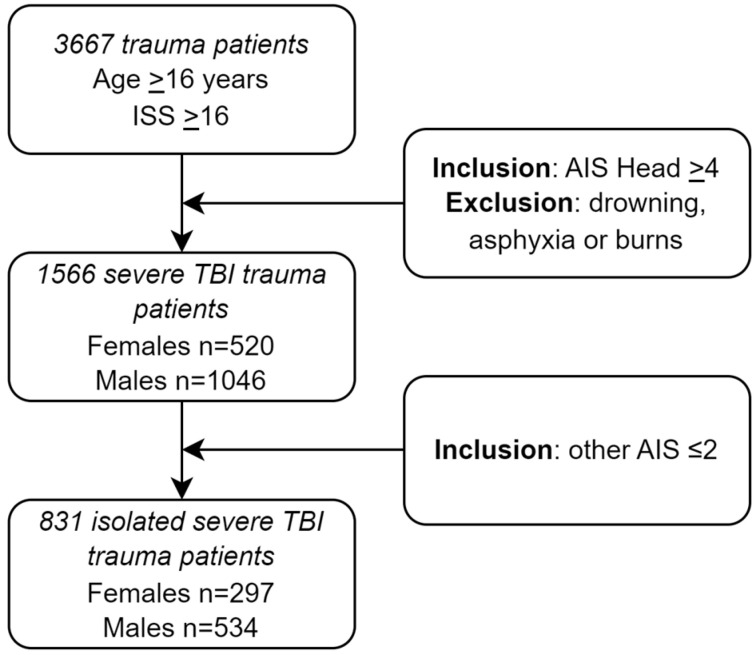
Flowchart of included trauma patients admitted to the ED of three level I trauma centers.

A total of 1566 severe TBI patients were included (Figure 1). The study population characteristics are presented in Table 1. Median age was higher in females compared to males (65 vs. 56 years, *p* < 0.001, Table 1). Patients aged 16 to 44 were more likely to be male (35.6% vs. 20.8%, *p* < 0.001, Table 1), whereas patients aged 45 or older where more likely to be female (79.2% vs. 64.4%, *p* < 0.001, Table 1). At ED admission, males compared to females showed a significantly higher ISS (26 vs. 25, *p* < 0.001, Table 1), lower RTS (5.97 vs. 5.97, *p* = 0.005, Table 1), higher SI (0.6 vs. 0.6, *p* = 0.012, Table 1), and lower GCS (6 vs. 8, *p* = 0.010, Table 1). 

Furthermore, males compared to females presented more frequently with AIS Spine ≥ 3 (6.6% vs. 4.0%, *p* = 0.041, Table 1), AIS Thorax ≥ 3 (26.5% vs. 17.3%, *p* < 0.001, Table 1), RTS < 4 (8.7% vs. 5.2%, *p* = 0.013, Table 1), SBP < 90 mmHg (8.4% vs. 4.8%, *p* = 0.009, Table 1), prehospital intubation (49.3% vs. 41.3%, *p* = 0.003), and P-HEMS assistance (36.3% vs. 27.9%, *p* < 0.001). Other patient characteristics did not show significant differences for sex.

### 3.2. Clinical Outcomes—Severe TBI

No significant differences in in-hospital mortality (34.2% vs. 33.3%, *p* = 0.704, Table 2), 30-day mortality (35.4% vs. 34.3%, *p* = 0.677, Table 2), or GOS ≤ 3 at hospital discharge (57.3% vs. 58.6%, *p* = 0.622, Table 2) were found for females vs. males, respectively (Table 2). Females compared to males did have a significantly lower ICU admission rate (65.2% vs. 72.5%, *p* = 0.003, Table 2), lower ICU LOS (3 vs. 2 days, *p* = 0.002, Table 2), and less frequently ICU LOS > 7 days (23.8% vs. 30.8%, *p* = 0.004, Table 2). Moreover, females compared to males showed shorter hospital LOS (6 vs. 7 days, *p* = 0.044, Table 2). Females were less frequently mechanically ventilated at ED admission (50.2% vs. 57.4%, *p* = 0.007, Table 2) and for a shorter period of time (1 vs. 2 days, *p* = 0.004, Table 2).

### 3.3. Logistic Regression Analysis—Severe TBI

Unadjusted logistic regression analysis showed a significant association between sex and the outcomes of ICU admission (OR 0.712; 95% CI 0.568–0.892, *p* = 0.003, Table 3), ICU LOS > 7 days (OR 0.704; 95% CI 0.553–0.896, *p* = 0.004, Table 3), and MV at ED admission (OR 0.749; 95% CI 0.607–0.925, *p* = 0.007, Table 3). Female sex was not associated with in-hospital mortality (OR 1.044; 95% CI 0.836–1.304, *p* = 0.704, Table 3), GOS ≤ 3 at hospital discharge (OR 1.056; 95% CI 0.850–1.312, *p* = 0.622, Table 3), or other outcome measures. After adjusting for covariates (Appendix A), female sex appears to be associated with ICU LOS > 7 days (OR 0.719; 95% CI 0.544–0.951, *p* = 0.021, Table 3) but not with ICU admission (OR 0.796; 95% CI 0.608–1.043, *p* = 0.098, Table 3) or MV at ED admission (OR 0.808; 95% CI 0.609–1.071, *p* = 0.139, Table 3).

### 3.4. Patient Characteristics—Severe TBI—Stratified for Age

Four hundred eighty patients aged 16 to 44 years were identified (22.5% females). Females compared to males were significantly younger (25 vs. 28 years, *p* = 0.001, Appendix B, Table A9), had lower SBP (122 vs. 130 mmHg, *p* = 0.002, Appendix B, Table A9), and lower SI (0.7 vs. 0.7, *p* = 0.038, Appendix B, Table A9) at ED admission. Other patient characteristics for the patients aged 16 to 44 years did not show significant differences for sex. 

In the ≥45 years group, 1086 patients were identified (37.9% female) of which females compared to males showed significantly higher age (70 vs. 68 years, *p* = 0.008, Appendix B, Table A9) and blunt injury as the mechanism of injury (99.8% vs. 97.8%, *p* = 0.008, Appendix B, Table A9). At ED admission, males compared to females showed a significantly higher ISS (25 vs. 25, *p* = 0.006, Appendix B, Table A9), lower RTS (5.97 vs. 6.76, *p* = 0.007, Appendix B, Table A9), higher SI (0.6 vs. 0.6, *p* = 0.008, Appendix B, Table A9), and lower GCS (7 vs. 9, *p* = 0.010, Appendix B, Table A9). Furthermore, males compared to females were more frequently presented with AIS Spine ≥ 3 (6.1% vs. 2.4%, *p* = 0.006, Appendix B, Table A9), AIS Thorax ≥ 3 (23.9% vs. 14.3%, *p* < 0.001, Appendix B, Table A9), SBP < 90 mmHg (8.3% vs. 3.6%, *p* = 0.003), prehospital intubation (46.7% vs. 37.2%, *p* = 0.002, Appendix B, Table A9), and P-HEMS assistance (36.5% vs. 26.0%, *p* = <0.001). In contrast, females more often underwent a craniotomy compared to males (22.1% vs. 16.8%, *p* = 0.029, Appendix B, Table A9). Other patient characteristics for the patients aged ≥ 45 years did not show significant differences for sex.

### 3.5. Clinical Outcomes—Severe TBI—Stratified for Age

No significant sex differences in outcomes in patients aged 16–44 years were found (Table 4). In patients aged ≥ 45 years, females compared to males were less often, (62.1% vs. 69.6%, *p* = 0.011, Table 4) and for shorter time periods, admitted to the ICU (2 vs. 2 days, *p* = 0.004, Table 4), and they showed lower rates of ICU LOS > 7 days (34.8% vs. 42.0%, *p* = 0.006, Table 4). Furthermore, females had a lower hospital LOS compared to their male counterparts (5 vs. 7, *p* = 0.024, Table 4). Also, females were less frequently mechanically ventilated at ED admission (46.6% vs. 53.4%, *p* = 0.029, Table 4) and for a shorter period of time (1 vs. 1 days, *p* = 0.005, Table 4). No significant differences in in-hospital mortality (37.6% vs. 38.9%, *p* = 0.681, Table 4), GOS ≤ 3 at hospital discharge (58.8% vs. 63.5%, *p* = 0.132, Table 4), or other outcomes were found for females vs. males, respectively (Table 4).

### 3.6. Logistic Regression Analysis—Severe TBI—Stratified for Age

No significant associations between sex and outcome were found in patients aged 16–44 years (Table 5). 

For patients aged ≥ 45 years, unadjusted logistic regression analysis showed a significant association between sex and the outcomes of ICU admission (OR 0.717; 95% CI 0.554–0.928, *p* = 0.012, Table 5), ICU LOS > 7 days (OR 0.667; 95% CI 0.501–0.889, *p* = 0.006, Table 5), and MV at ED admission (OR 0.761; 95% CI 0.595–0.973, *p* = 0.030, Table 5). Female sex was not associated with in-hospital mortality (OR 0.948; 95% CI 0.737–1.221, *p* = 0.681, Table 5), GOS ≤ 3 at hospital discharge (OR 1.217; 95% CI 0.942–1.572, *p* = 0.133, Table 5), or other outcome measures. After adjusting for covariates, female sex was still associated with ICU LOS > 7 days (OR 0.644; 95% CI 0.462–0.898, *p* = 0.010, Table 5) and with hospital LOS > 7 days (OR 0.755; 95% CI 0.580–0.983, *p* = 0.037, Table 5), but not with ICU admission (OR 0.742; 95% CI 0.544–1.012, *p* = 0.060, Table 5) or MV at ED admission (OR 0.761; 95% CI 0.547–1.058, *p* = 0.104, Table 5).

### 3.7. Patient Characteristics—Isolated Severe TBI 

Eight hundred thirty-one isolated severe TBI patients aged ≥ 16 years with an AIS Head ≥ 4 and all other AIS scores ≤ 2 were admitted (Figure 1). Median age was higher in females compared to males (68 vs. 58 years, *p* < 0.001, Appendix B, Table A10). Patients aged 16 to 44 where more likely to be male (32.2% vs. 13.5%, *p* < 0.001, Appendix B, Table A10), whereas patients aged 45 or older where more likely to be female (86.5% vs. 67.8%, *p* < 0.001, Appendix B, Table A10). A blunt mechanism of injury was more frequent in females compared to males (99.7% vs. 97.2%, *p* = 0.013, Appendix B, Table A10). Other patient characteristics did not show significant differences for sex.

### 3.8. Clinical Outcomes—Isolated Severe TBI 

No significant sex differences in clinical outcomes were found (Appendix B, Table A11).

### 3.9. Logistic Regression Analysis—Isolated Severe TBI 

Unadjusted as well as adjusted logistic regression analysis, respectively, showed no significant association between sex and the outcomes (Appendix B, Table A12). 

### 3.10. Patient Characteristics—Isolated Severe TBI—Stratified for Age

Two hundred twelve patients aged 16 to 44 years were identified (18.9% females). Females compared to males were significantly younger (25 vs. 30 years, *p* = 0.014, Appendix B, Table A13) and had lower SBP (125 vs. 134 mmHg, *p* = 0.005, Appendix B, Table A13) at ED admission. Other patient characteristics for the patients aged 16 to 44 years did not show significant differences for sex. 

In the ≥45 years group, 619 patients were identified (41.5% female) of which females compared to males showed significantly higher age (71 vs. 68 years, *p* = 0.024, Appendix B, Table A13) and blunt injury as the mechanism of injury (99.2% vs. 96.6%, *p* = 0.003, Appendix B, Table A13). Other patient characteristics for the patients aged ≥ 45 years did not show significant differences for sex. 

### 3.11. Clinical Outcomes—Isolated Severe TBI—Stratified for Age

No significant sex differences (in both age groups, 16–44 and ≥45 years, respectively) in clinical outcomes were found (Appendix B, Table A14).

### 3.12. Logistic Regression Analysis—Isolated Severe TBI—Stratified for Age

Unadjusted as well as adjusted logistic regression analysis in both age groups, 16–44 and ≥45 years, showed no significant association between sex and outcomes (Appendix B, Table A15). 

## 4. Discussion

This study investigated sex differences in outcomes in a contemporary population of severe TBI patients at level 1 trauma centers in the Netherlands in a mature trauma system with complete datasets. In severe TBI patients, female sex compared to male sex was associated with lower ICU admission rates, shorter ICU LOS, shorter hospital LOS, and less frequent and shorter MV in crude analysis (Table 2). Furthermore, when adjusting for a priori-determined covariates in severe TBI patients, female sex appears to be associated with a decreased (28.1%) likelihood for ICU LOS > 7 days compared to male sex (Table 3). Significant associations between sex and in-hospital mortality, GOS ≤ 3 at discharge, and 30-day mortality could not be confirmed. Subgroup analysis for age in severe TBI patients showed that the above mentioned crude sex differences in outcomes favoring females seem to be limited to the older patient group (≥5 years) (Table 4). When adjusting for covariates in these older aged severe TBI patients, female sex compared to male sex appears to be associated with a decreased (18% and 7.2%) likelihood for ICU LOS > 7 days and hospital LOS > 7 days, respectively (Table 5). In severe isolated TBI patients, an association between sex and clinical outcomes could not be confirmed. Subgroup analysis for age in severe isolated TBI similarly did not show sex differences in patient outcomes. 

This study specifically focused on trauma patients with severe TBI as defined by Head AIS ≥ 4. According to our knowledge, this is the first study regarding sexual dimorphism in outcomes in patients with severe TBI as defined by Head AIS ≥ 4. In contrast to other studies, a Head AIS ≥ 4 was an inclusion criterion in this study instead of GCS, as a low GCS may also include intubated patients without TBI [7,11,13]. 

In line with our earlier-mentioned findings regarding the association between sex and outcome in severe TBI, Mikolic et al. found that, following moderate/severe TBI, females compared to males had shorter median hospital LOS and mild TBI females were less likely to be admitted to the ICU [11]. Sex differences in the Glasgow Outcome Scale Extended (GOSE) for females with mild TBI and more post-concussion symptoms for females after suffering from moderate/severe TBI were found [11], whereas in our study sex differences in GOS were not found. However, our follow-up may not have been long enough to find a significant difference, as patients with TBI usually still recover over time, both during rehabilitation and after hospital discharge. Similar to our findings regarding mortality, Mikolic et al., Yeung et al., Leitgeb et al., and Ponsford et al. did not find a significant association between sex and mortality following TBI [10,11,12,13]. Contrary to our findings, Berry et al., Davis et al., and De Guise et al. did find a survival advantage for females compared to males from the same age group [5,6,7]. 

In the literature, several studies report on the role of age in the association between sex and outcome. Similar to our finding that females (aged ≥ 45 years) had lower ICU admission rates and shorter ICU/hospital LOS, multiple studies into sex differences in severe (non TBI) trauma patients also demonstrated that males have a significantly higher rate of ICU admission and a longer ICU LOS compared to females [23,24,25]. De Guise et al. found no sex differences in hospital LOS nor in GOSE outcome [7]. Furthermore, Berry et al. included 72,294 patients with moderate/severe TBI (AIS Head ≥ 3) and applied stratification for age as a surrogate for hormonal status: 14–45 years (premenopausal), 46–55 years (perimenopausal), and >55 years (postmenopausal). In line with our findings, the study did not find differences in mortality for premenopausal women compared to age-matched males, but, contrarily, it did find a significantly lower risk for mortality for perimenopausal/postmenopausal females [5]. Likewise, Davis et al. found a lower mortality for females aged ≥ 50 years compared to age-matched males in patients with moderate-to-severe TBI (AIS Head ≥ 3) [6] and De Guise et al. found that female sex was associated with a reduced likelihood of in-hospital mortality after adjusting for covariates in patients with moderate/severe TBI [7]. Interestingly, Czosnyka et al. and Ottochian et al. found not lower, but higher mortality rates for females compared to males [8,9]. Czosnyka et al. showed that premenopausal females (<50 years) had a significantly higher mortality rate compared to males [8] and Ottochian et al. that female postmenopausal (≥55 years) patients with isolated moderate-to-severe TBI (AIS Head ≥ 3) had a significantly higher mortality rate [9]. Critically reviewing the existing literature and the results from this study, it appears that the baseline characteristics from the (older) severe TBI patients seemed to differ more in comparison to the other researched populations. Notably, a craniotomy was significantly more often performed in females aged > 45 years than in their male counterparts, which might suggest that females were more likely to have treatable injuries. Interestingly, although one could argue that males seem more severely injured compared to females, potentially explaining sex differences in outcomes such as ICU admission rate and ICU/hospital LOS, this does not definitively explain the similar mortality rates and GOS found in females and males. 

It is important to evaluate the factors which could possibly contribute to observed sex differences in outcomes after TBI. Farace and Alves [26] performed a meta-analysis researching sex differences in TBI outcomes. They identified several contributing factors, e.g., mechanism of the accident (females are more likely to use safety restraints, which could change the site of impact/increase the severity of the accident to cause injury), mechanism in the brain (sex differences in functional organization of the brain are present), treatment effects (sex-related differences in brain metabolism are present), and premorbid sex differences (sex differences in incidence of TBI are broadly known, with males being more often affected). Other covariates could be of influence and mediate the effect between sex and outcome, such as injury severity, mechanism of injury, pre-existing comorbidity, and so on. Also, a speculative explanation of contradictory findings in the existing literature could be that unknown mediators attribute to the found effect, such as hormonal status, with estradiol or testosterone having a positive or negative effect on the association between sex and outcome. Unfortunately, studies regularly fail to measure hormone levels, which could potentially explain the sex differences in outcomes. There is, though, one prospective cohort study which measured sex steroids during ICU stays in severely injured patients and determined the association with mortality [27]. The authors did not find a sex difference in mortality but did find elevated estradiol levels in non-survivors regardless of sex. Therefore, estradiol may play a bigger role in relation to outcomes in the severely injured trauma patient than we are currently aware of [27]. In contrast, preclinical studies in rodents show a protective effect of administered estrogen in mortality rates following TBI [14,15,16,17,18]. These findings suggest higher levels of estrogen being potentially beneficial for survival after TBI. It is important to state that, since the current study did not measure hormonal levels during hospital admission, we can only speculate about hormones playing a role in the association between sex and outcome.

There are several limitations in this retrospective study. Females represent only 33.2% of the whole study population. Nevertheless, comparable percentages of included females compared to males were seen in similar studies [5,6,7,11]. And, despite the relatively large included population, subgroup sizes may be too small to detect a (age effect on) survival advantage for female patients with severe (isolated) TBI compared to male patients. Notably, other studies included more than 10,000 patients [6,9]. However, those articles also included moderate TBI whereas the current study only included patients with severe TBI, potentially resulting in different outcomes caused by the larger extent of damage to the brain. Because of the retrospective nature of the Trauma Registry, it was impossible to collect data on reproductive cycles, hormonal status, hormone replacements, or oral contraceptives. Therefore, patients were divided in subgroups as a surrogate for hormonal status: premenopausal or hormonally active phase (aged 16–44 years), in which estrogen levels are considered highest, and both the peri- and postmenopausal phase (aged ≥ 45 years), in which hormonal status is unclear or most likely inactive. Regarding parameter outcomes, GOS at hospital discharge did not show a significant sex difference. However, our follow-up may not have been long enough to find a significant difference, as patients with TBI usually still recover over time, during rehabilitation and after hospital discharge. This study did not further investigate different trauma mechanisms (e.g., fall from height or high velocity vehicle accident) or the use of direct oral anticoagulants, vitamin-k antagonists or thrombocyte aggregation inhibitors, which may lead to differences in outcomes after severe TBI. Furthermore, unknown factors may remain, contributing to the found sex differences in outcomes in the current study. Also, results regarding the association between sex and secondary outcomes serve an exploratory purpose and should therefore be seen as a means/focus for future studies and not to draw strong conclusions from. Lastly, it should be stated that this investigation adopts an exploratory approach, thereby precluding any conclusive assertion of causality.

One of the strengths of this study is, with regard to sample size, the relatively large included population: 1566 patients with severe TBI, of which 831 patients had severe isolated TBI. Other strengths of this study include the exclusion of trauma patients with drowning, asphyxia, or burns to reduce heterogeneity within the population due to difference in pathophysiology and the complete data on patient characteristics, treatment, and outcome, as well as the multicentered nature of this study. Lastly, in the adjusted outcomes reported in this study, the a priori-determined covariates method was used.

This study provides insights into the association between sex and outcomes in trauma patients with severe TBI. Sex differences in outcomes following severe TBI are found, potentially favoring (older) females. This suggests some support for the hypothesis that female compared to male trauma patients might benefit from protective effects, but the conclusion that females are better able to withstand trauma and sustain a physiologic response after severe TBI remains to be elucidated. Future research may focus on large prospective studies including additional variables such as hormonal status (including hormone serum levels), exact mechanism of injury, and use of hormone replacement therapy, direct oral anticoagulants, vitamin-k antagonists and thrombocyte aggregation inhibitors. This information will help to unravel sex differences in outcomes after trauma, specifically in severe TBI, and may eventually lead to future improvements of patient-specific treatment.

## 5. Conclusions

This study found sex differences in outcomes following severe TBI, potentially favoring females. In trauma patients with severe TBI, especially ≥ 45 years of age, female sex compared to male sex was significantly associated with lower ICU admission rates, shorter ICU LOS, shorter hospital LOS, and less frequent and shorter MV after crude analysis. Furthermore, female sex compared to male sex in severe TBI patients appears, after adjusting for covariates, significantly associated with shorter ICU LOS and hospital LOS. On the contrary, significant associations between sex and in-hospital mortality, GOS ≤ 3 at discharge, and 30-day mortality could not be confirmed in this study. 

Even after stratification for age, no sex differences in outcomes in severe isolated TBI patients were found. The current results suggest that female sex might be related to different physiological responses to severe TBI. Nevertheless, causality, pathophysiology, and associations with outcomes such as mortality should be investigated in future prospective studies with larger populations to help unravel sex differences in outcomes after trauma. 

## Figures and Tables

**Table 1:** Patient characteristics in the study population of trauma patients with severe TBI.

	Total (*n* = 1566)	Female (*n* = 520)	Male (*n* = 1046)	*p*-Value
Age				
Age (years) median (IQR)	60 (16–98)	65 (16–98)	56 (16–95)	<0.001
Age 16–44 years	480 (30.7%)	108 (20.8%)	372 (35.6%)	<0.001
Age ≥ 45 years	1086 (69.3%)	412 (79.2%)	674 (64.4%)	<0.001
Injury and severity				
Blunt	1526 (98.0%)	511 (98.0%)	1015 (97.6%)	0.098
Penetrating	31 (2.0%)	6 (2.0%)	25 (2.4%)	0.098
AIS Neck ≥ 3	17 (1.1%)	3 (0.6%)	14 (1.3%)	0.171
AIS Spine ≥ 3	90 (5.7%)	21 (4.0%)	69 (6.6%)	0.041
AIS Thorax ≥ 3	367 (23.4%)	90 (17.3%)	277 (26.5%)	<0.001
AIS Abdomen ≥ 3	42 (2.7%)	13 (2.5%)	29 (2.8%)	0.753
AIS Lower extremities ≥ 3	110 (7.0%)	35 (6.7%)	75 (7.2%)	0.749
ISS median (IQR)	26 (16–75)	25 (16–75)	26 (16–75)	<0.001
Vital signs				
RTS median (IQR)	5.97 (0–7.84)	5.97 (0–7.84)	5.97 (0–7.84)	0.005
RTS < 4	118 (7.5%)	27 (5.2%)	91 (8.7%)	0.013
SBP median (IQR)	137 (45–245)	138 (45–245)	136 (50–240)	0.156
SBP < 90 mmHg	113 (7.2%)	25 (4.8%)	88 (8.4%)	0.009
SI median (IQR)	0.6 (0.2–2.7)	0.6 (0.2–2.3)	0.6 (0.2–2.7)	0.012
Neurological				
GCS median (IQR)	6 (3–15)	8 (3–15)	6 (3–15)	0.010
GCS ≤ 8	829 (52.9%)	264 (50.8%)	565 (54.0%)	0.226
Craniotomy	300 (19.2%)	111 (21.3%)	189 (18.1%)	0.121
ICP measurement	179 (11.4%)	48 (9.2%)	131 (12.5%)	0.054
Comorbidity				
Healthy or mild (ASA ≤ II)	1077 (68.7%)	369 (71.0%)	708 (67.7%)	0.911
Severe comorbidity (ASA ≥ III)	289 (18.5%)	98 (18.8%)	191 (18.3%)	0.911
Prehospital				
Prehospital intubation	720 (46.7%)	211 (41.3%)	509 (49.3%)	0.003
P-HEMS	525 (33.5%)	145 (27.9%)	380 (36.3%)	<0.001

AIS, Abbreviated Injury Scale; ASA, American Society of Anesthesiologists; GCS, Glasgow Coma Scale; ICP, Intracranial pressure; IQR, Interquartile range; ISS, Injury Severity Score; P-HEMS, Physician-staffed helicopter emergency medical services; RTS, Revised Trauma Score; SBP, Systolic blood pressure; SI, Shock index.

**Table 2:** Clinical outcomes of trauma patients with severe TBI stratified for sex.

	Total (*n* = 1566)	Female (*n* = 520)	Male (*n* = 1046)	*p*-Value
Primary outcomes				
In-hospital mortality	526 (33.6%)	178 (34.2%)	348 (33.3%)	0.704
GOS ≤ 3 at discharge	879 (58.2%)	287 (57.3%)	592 (58.6%)	0.622
ICU admission	1097 (70.1%)	339 (65.2%)	758 (72.5%)	0.003
Secondary outcomes				
30-day mortality	543 (34.7%)	184 (35.4%)	359 (34.3%)	0.677
ICU days median (IQR)	2 (0–67)	2 (0–67)	3 (0–67)	0.002
ICU > 7 days	446 (28.5%)	124 (23.8%)	322 (30.8%)	0.004
Hospital days median (IQR)	6 (0–152)	6 (0–152)	7 (0–135)	0.044
Hospital > 7 days	761 (48.6%)	236 (45.4%)	525 (50.2%)	0.073
MV admission	861 (55.0%)	261 (50.2%)	600 (57.4%)	0.007
MV days median (IQR)	1 (0–60)	1 (0–37)	2 (0–60)	0.004
MV > 7 days	334 (38.8%)	105 (40.2%)	229 (38.2%)	0.439

GOS, Glasgow Outcome Score; ICU, Intensive Care Unit; IQR, Interquartile range; MV, Mechanical ventilation.

**Table 3:** The association of female sex with clinical outcome parameters in trauma patients presenting with severe TBI.

	OR	95% CI	*p*-Value
Unadjusted values			
In-hospital mortality	1.044	0.836–1.304	0.704
GOS ≤ 3 at discharge	1.056	0.850–1.312	0.622
ICU admission	0.712	0.568–0.892	0.003
30-day mortality	1.048	0.841–1.306	0.677
ICU > 7 days	0.704	0.553–0.896	0.004
Hospital > 7 days	0.825	0.668–1.018	0.073
MV admission	0.749	0.607–0.925	0.007
MV > 7 days	0.903	0.696–1.170	0.439
Adjusted values *			
In-hospital mortality	1.074	0.822–1.405	0.600
GOS ≤ 3 at discharge	1.127	0.857–1.481	0.394
ICU admission	0.796	0.608–1.043	0.098
30-day mortality	1.051	0.803–1.375	0.717
ICU > 7 days	0.719	0.544–0.951	0.021
Hospital > 7 days	0.837	0.667–1.049	0.122
MV admission	0.808	0.609–1.071	0.139
MV > 7 days	1.034	0.765–1.396	0.828

* corrected for the a priori-determined covariates age, MOI, ISS, RTS < 4, GCS ≤ 8, SI, ASA ≥ III, ICP, and craniotomy (Appendix A). ASA, American Society of Anesthesiologists; GCS, Glasgow Coma Scale; GOS, Glasgow Outcome Score; ICP, Intracranial pressure; ICU, Intensive Care Unit; ISS, Injury Severity Score; MOI, Mechanism of Injury; MV, Mechanical ventilation; RTS, Revised Trauma Score; SI, Shock index.

**Table 4:** Clinical outcomes of trauma patients with severe TBI stratified for age 16–44 years and ≥45 years.

	16–44 Years			≥45 Years		
	Female (*n* = 108)	Male (*n* = 372)	*p*-Value	Female (*n* = 412)	Male (*n* = 674)	*p*-Value
Primary outcomes						
In-hospital mortality	23 (21.3%)	86 (23.1%)	0.691	155 (37.6%)	262 (38.9%)	0.681
GOS ≤ 3 at discharge	54 (51.4%)	178 (49.7%)	0.758	233 (58.8%)	414 (63.5%)	0.132
ICU admission	83 (76.9%)	289 (77.7%)	0.855	256 (62.1%)	469 (69.6%)	0.011
Secondary outcomes						
30-day mortality	23 (21.3%)	85 (22.8%)	0.734	161 (39.1%)	274 (40.7%)	0.607
ICU days median (IQR)	3 (0–67)	3 (0–59)	0.908	2 (0–38)	2 (0–67)	0.004
ICU > 7 days	35 (42.2%)	125 (43.3%)	0.817	89 (34.8%)	197 (42.0%)	0.006
Hospital days median (IQR)	8 (0–152)	7 (0–108)	0.431	5 (0–96)	7 (0–135)	0.024
Hospital > 7 days	55 (50.9%)	189 (50.8%)	0.983	181 (43.9%)	336 (49.9%)	0.058
MV admission	69 (63.9%)	240 (64.5%)	0.905	192 (46.6%)	360 (53.4%)	0.029
MV days median (IQR)	2 (0–37)	2 (0–42)	0.606	1 (0–33)	1 (0–60)	0.005
MV > 7 days	32 (46.4%)	92 (38.3%)	0.306	73 (38.0%)	137 (38.1%)	0.291

GOS, Glasgow Outcome Score; ICU, Intensive Care Unit; IQR, Interquartile range; MV, Mechanical ventilation.

**Table 5:** The association of female sex with clinical outcome parameters in trauma patients presenting with severe TBI stratified for age 16–44 years and ≥45 years.

	16–44 Years			≥45 Years		
	OR	95% CI	*p*-Value	OR	95% CI	*p*-Value
Unadjusted values						
In-hospital mortality	0.900	0.535–1.513	0.691	0.948	0.737–1.221	0.681
GOS ≤ 3 at discharge	0.934	0.604–1.443	0.758	1.217	0.942–1.572	0.133
ICU admission	0.953	0.573–1.587	0.855	0.717	0.554–0.928	0.012
30-day mortality	0.914	0.543–1.537	0.734	0.936	0.729–1.203	0.607
ICU > 7 days	0.947	0.600–1.496	0.817	0.667	0.501–0.889	0.006
Hospital > 7 days	1.005	0.655–1.542	0.983	0.788	0.616–1.008	0.058
MV admission	0.973	0.623–1.521	0.905	0.761	0.595–0.973	0.030
MV > 7 days	1.281	0.797–2.062	0.307	0.844	0.616–1.156	0.291
Adjusted values *						
In-hospital mortality	0.818	0.426–1.571	0.546	1.132	0.836–1.534	0.422
GOS ≤ 3 at discharge	1.029	0.578–1.833	0.922	1.192	0.867–1.639	0.280
ICU admission	0.773	0.416–1.435	0.415	0.742	0.544–1.012	0.060
30-day mortality	0.858	0.449–1.640	0.644	1.090	0.804–1.478	0.578
ICU > 7 days	0.820	0.471–1.429	0.484	0.644	0.462–0.898	0.010
Hospital > 7 days	0.928	0.575–1.498	0.760	0.755	0.580–0.983	0.037
MV admission	0.680	0.363–1.273	0.228	0.761	0.547–1.058	0.104
MV > 7 days	1.318	0.749–2.321	0.338	0.933	0.647–1.345	0.709

* corrected for the a priori-determined covariates age, MOI, ISS, RTS < 4, GCS ≤ 8, SI, ASA ≥ III, ICP, and craniotomy. ASA, American Society of Anesthesiologists; GCS, Glasgow Coma Scale; GOS, Glasgow Outcome Score; ICP, Intracranial pressure; ICU, Intensive Care Unit; ISS, Injury Severity Score; MOI, Mechanism of Injury; MV, Mechanical ventilation; RTS, Revised Trauma Score; SI, Shock index.

## Data Availability

The datasets generated and analyzed during the current study are available from the corresponding author on reasonable request.

## Appendix A

**Table A1:** Multivariable logistic regression analysis of in-hospital mortality in trauma patients presenting with severe TBI.

In-Hospital Mortality								
	B	S.E.	Wald	df	Sig.	Exp(B)	95% C.I. for EXP(B)	
							Lower	Upper
Sex	0.072	0.137	0.274	1	0.600	1.074	0.822	1.405
Age	0.045	0.004	136.489	1	<0.001	1.046	1.038	1.054
MOI	1.037	0.477	4.723	1	0.030	2.822	1.107	7.192
ISS	0.056	0.007	59.276	1	<0.001	1.058	1.043	1.073
RTS < 4	0.537	0.265	4.115	1	0.043	1.711	1.018	2.876
GCS ≤ 8	1.774	0.149	141.624	1	<0.001	5.897	4.403	7.898
SI	1.027	0.246	17.479	1	<0.001	2.793	1.726	4.522
ASA ≥ III	0.270	0.170	2.529	1	0.112	1.310	0.939	1.826
ICP	−0.132	0.199	0.441	1	0.506	0.876	0.593	1.294
Craniotomy	0.239	0.165	2.100	1	0.147	1.270	0.919	1.753
Constant	−6.870	0.415	274.263	1	<0.001	0.001		

**Table A2:** Multivariable logistic regression analysis of GOS ≤ 3 at discharge in trauma patients presenting with severe TBI.

GOS ≤ 3 at Discharge								
	B	S.E.	Wald	df	Sig.	Exp(B)	95% C.I. for EXP(B)	
							Lower	Upper
Sex	0.119	0.140	0.728	1	0.394	1.127	0.857	1.481
Age	−0.035	0.004	92.288	1	<0.001	0.966	0.959	0.973
MOI	−0.938	0.513	3.340	1	0.068	0.391	0.143	1.070
ISS	−0.067	0.009	61.050	1	<0.001	0.936	0.920	0.951
RTS < 4	0.045	0.327	0.019	1	0.891	1.046	0.551	1.986
GCS ≤ 8	−2.071	0.149	193.604	1	<0.001	0.126	0.094	0.169
SI	−1.026	0.295	12.060	1	<0.001	0.358	0.201	0.640
ASA ≥ III	−0.741	0.178	17.388	1	<0.001	0.476	0.336	0.675
ICP	−0.827	0.228	13.104	1	<0.001	0.437	0.279	0.684
Craniotomy	−0.844	0.170	24.538	1	<0.001	0.430	0.308	0.600
Constant	5.479	0.397	190.591	1	<0.001	239.649		

**Table A3:** Multivariable logistic regression analysis of ICU admission in trauma patients presenting with severe TBI.

ICU Admission								
	B	S.E.	Wald	df	Sig.	Exp(B)	95% C.I. for EXP(B)	
							Lower	Upper
Sex	−0.228	0.138	2.746	1	0.098	0.796	0.608	1.043
Age	−0.017	0.003	24.535	1	<0.001	0.984	0.977	0.990
MOI	0.076	0.470	0.026	1	0.872	1.079	0.430	2.710
ISS	0.037	0.008	21.033	1	<0.001	1.038	1.021	1.054
RTS < 4	−0.190	0.309	0.380	1	0.538	0.827	0.451	1.515
GCS ≤ 8	1.248	0.146	73.219	1	<0.001	3.484	2.618	4.638
SI	−0.395	0.265	2.218	1	0.136	0.674	0.401	1.133
ASA ≥ III	0.163	0.167	0.953	1	0.329	1.177	0.848	1.633
ICP	3.554	0.719	24.439	1	<0.001	34.952	8.542	143.024
Craniotomy	2.158	0.247	76.113	1	<0.001	8.656	5.330	14.057
Constant	0.144	0.313	0.212	1	0.645	1.155		

**Table A4:** Multivariable logistic regression analysis of 30-day mortality in trauma patients presenting with severe TBI.

30-Day Mortality								
	B	S.E.	Wald	df	Sig.	Exp(B)	95% C.I. for EXP(B)	
							Lower	Upper
Sex	0.050	0.137	0.132	1	0.717	1.051	0.803	1.375
Age	0.047	0.004	147.664	1	<0.001	1.048	1.040	1.056
MOI	1.025	0.485	4.467	1	0.035	2.787	1.077	7.212
ISS	0.054	0.007	54.834	1	<0.001	1.056	1.041	1.071
RTS <4	0.514	0.267	3.705	1	0.054	1.672	0.991	2.822
GCS ≤ 8	1.878	0.151	155.285	1	<0.001	6.541	4.868	8.789
SI	1.027	0.247	17.254	1	<0.001	2.794	1.721	4.537
ASA ≥ III	0.472	0.170	7.758	1	0.005	1.604	1.150	2.236
ICP	−0.221	0.200	1.215	1	0.270	0.802	0.541	1.188
Craniotomy	0.241	0.165	2.127	1	0.145	1.273	0.920	1.760
Constant	−6.968	0.419	277.091	1	<0.001	0.001		

**Table A5:** Multivariable logistic regression analysis of ICU > 7 days in trauma patients presenting with severe TBI.

ICU > 7 Days								
	B	S.E.	Wald	df	Sig.	Exp(B)	95% C.I. for EXP(B)	
							Lower	Upper
Sex	−0.330	0.143	5.355	1	0.021	0.719	0.544	0.951
Age	−0.006	0.003	3.328	1	0.068	0.994	0.988	1.000
MOI	0.052	0.459	0.013	1	0.910	1.054	0.428	2.593
ISS	0.031	0.007	20.960	1	<0.001	1.031	1.018	1.045
RTS < 4	−0.960	0.288	11.082	1	<0.001	0.383	0.218	0.674
GCS ≤ 8	1.027	0.143	51.640	1	<0.001	2.794	2.111	3.697
SI	−0.455	0.252	3.271	1	0.071	0.634	0.387	1.039
ASA ≥ III	0.335	0.182	3.404	1	0.065	1.398	0.979	1.997
ICP	2.180	0.193	127.288	1	<0.001	8.846	6.058	12.919
Craniotomy	1.461	0.151	94.142	1	<0.001	4.311	3.209	5.791
Constant	−2.328	0.319	53.408	1	<0.001	0.097		

**Table A6:** Multivariable logistic regression analysis of Hospital > 7 days in trauma patients presenting with severe TBI.

Hospital > 7 Days								
	B	S.E.	Wald	df	Sig.	Exp(B)	95% C.I. for EXP(B)	
							Lower	Upper
Sex	−0.178	0.115	2.395	1	0.122	0.837	0.667	1.049
Age	−0.005	0.003	4.002	1	0.045	0.995	0.989	1.000
MOI	−0.307	0.400	0.590	1	0.442	0.736	0.336	1.610
ISS	0.011	0.006	3.363	1	0.067	1.011	0.999	1.022
RTS < 4	−0.719	0.248	8.372	1	0.004	0.487	0.299	0.793
GCS ≤ 8	0.012	0.118	0.011	1	0.917	1.012	0.803	1.276
SI	−0.594	0.218	7.445	1	0.006	0.552	0.360	0.846
ASA ≥ III	0.055	0.146	0.143	1	0.706	1.057	0.793	1.408
ICP	1.539	0.194	62.802	1	<0.001	4.661	3.185	6.820
Craniotomy	0.978	0.140	48.802	1	<0.001	2.659	2.021	3.499
Constant	0.093	0.258	0.129	1	0.720	1.097		

**Table A7:** Multivariable logistic regression analysis of MV admission in trauma patients presenting with severe TBI.

MV Admission								
	B	S.E.	Wald	df	Sig.	Exp(B)	95% C.I. for EXP(B)	
							Lower	Upper
Sex	−0.214	0.144	2.194	1	0.139	0.808	0.609	1.071
Age	−0.014	0.003	17.592	1	<0.001	0.986	0.979	0.992
MOI	1.241	0.517	5.751	1	0.016	3.459	1.254	9.538
ISS	0.038	0.008	24.373	1	<0.001	1.039	1.023	1.055
RTS < 4	−0.195	0.288	0.460	1	0.498	0.823	0.468	1.447
GCS ≤ 8	2.207	0.146	227.996	1	<0.001	9.086	6.823	12.100
SI	−0.338	0.263	1.649	1	0.199	0.713	0.426	1.194
ASA ≥ III	0.451	0.179	6.303	1	0.012	1.569	1.104	2.231
ICP	2.737	0.354	59.744	1	<0.001	15.434	7.711	30.891
Craniotomy	2.034	0.187	117.747	1	<0.001	7.644	5.294	11.037
Constant	−1.520	0.321	22.375	1	<0.001	0.219		

**Table A8:** Multivariable logistic regression analysis of MV > 7 days in trauma patients presenting with severe TBI.

MV >7 Days								
	B	S.E.	Wald	df	Sig.	Exp(B)	95% C.I. for EXP(B)	
							Lower	Upper
Sex	0.033	0.153	0.047	1	0.828	1.034	0.765	1.396
Age	−0.008	0.004	4.509	1	0.034	0.992	0.985	0.999
MOI	−0.134	0.523	0.066	1	0.798	0.875	0.314	2.436
ISS	0.033	0.007	21.238	1	<0.001	1.033	1.019	1.048
RTS < 4	−0.660	0.302	4.784	1	0.029	0.517	0.286	0.934
GCS ≤ 8	1.195	0.165	52.379	1	<0.001	3.303	2.390	4.565
SI	−0.456	0.272	2.806	1	0.094	0.634	0.372	1.081
ASA ≥ III	0.245	0.206	1.413	1	0.235	1.278	0.853	1.914
ICP	2.074	0.191	117.374	1	<0.001	7.957	5.467	11.579
Craniotomy	1.522	0.164	86.072	1	<0.001	4.580	3.321	6.316
Constant	−3.046	0.359	71.842	1	<0.001	0.048		

## Appendix B

**Table A9:** Patient characteristics in the study population of trauma patients with severe TBI stratified for age 16–44 years and ≥45 years.

	16–44 Years			≥45 Years		
	Female (*n* = 108)	Male (*n* = 372)	*p*-Value	Female (*n* = 412)	Male (*n* = 674)	*p*-Value
Age						
Age (years) median (IQR)	25 (16–44)	28 (16–44)	0.001	70 (45–98)	68 (45–95)	0.008
Injury and severity						
Blunt	103 (95.4%)	361 (97.3%)	0.345	408 (99.8%)	654 (97.8%)	0.008
Penetrating	5 (4.6%)	10 (2.7%)	0.345	1 (0.2%)	15 (2.2%)	0.008
AIS Neck ≥ 3	1 (0.9%)	9 (2.4%)	0.339	2 (0.5%)	5 (0.7%)	0.716
AIS Spine ≥ 3	11 (10.2%)	28 (7.5%)	0.373	10 (2.4%)	41 (6.1%)	0.006
AIS Thorax ≥ 3	31 (28.7%)	116 (31.2%)	0.623	59 (14.3%)	161 (23.9%)	<0.001
AIS Abdomen ≥ 3	11 (10.2%)	23 (6.2%)	0.153	2 (0.5%)	6 (0.9%)	0.717
AIS Lower extremities ≥ 3	17 (15.7%)	40 (10.8%)	0.158	18 (4.4%)	35 (5.2%)	0.541
ISS median (IQR)	26 (16–75)	29 (16–75)	0.395	25 (16–59)	25 (16–75)	0.006
Vital signs						
RTS median (IQR)	4.09 (0–7.84)	5.03 (0–7.84)	0.650	6.76 (0–7.84)	5.97 (1.76–7.84)	0.007
RTS < 4	9 (8.3%)	45 (12.1%)	0.276	18 (4.4%)	46 (6.8%)	0.095
SBP median (IQR)	122 (54–184)	130 (50–240)	0.002	145 (45–245)	141 (50–240)	0.113
SBP < 90 mmHg	10 (9.3%)	32 (8.6%)	0.832	15 (3.6%)	56 (8.3%)	0.003
SI median (IQR)	0.7 (0.4–2.2)	0.7 (0.2–2.7)	0.038	0.6 (0.2–2.3)	0.6 (0.2–2.4)	0.008
Neurological						
GCS median (IQR)	3 (3–15)	3 (3–15)	0.610	9 (3–15)	7 (3–15)	0.010
GCS ≤ 8	70 (64.8%)	220 (59.1%)	0.288	194 (47.1%)	345 (51.2%)	0.190
Craniotomy	20 (18.5%)	76 (20.4%)	0.662	91 (22.1%)	113 (16.8%)	0.029
ICP measurement	18 (16.7%)	60 (16.1%)	0.894	30 (7.3%)	71 (10.5%)	0.073
Comorbidity						
Healthy or mild (ASA ≤ II)	93 (97.9%)	294 (95.1%)	0.381	276 (74.2%)	414 (70.2%)	0.177
Severe comorbidity (ASA ≥ III)	2 (2.1%)	15 (4.9%)	0.381	96 (25.8%)	176 (29.8%)	0.177
Prehospital						
Prehospital intubation	60 (57.1%)	198 (54.1%)	0.581	151 (37.2%)	311 (46.7%)	0.002
P-HEMS	38 (35.2%)	134 (36.0%)	0.873	107 (26.0%)	246 (36.5%)	<0.001

AIS, Abbreviated Injury Scale; ASA, American Society of Anesthesiologists; GCS, Glasgow Coma Scale; ICP, Intracranial pressure; IQR, Interquartile range; ISS, Injury Severity Score; P-HEMS, Physician-staffed helicopter emergency medical services; RTS, Revised Trauma Score; SBP, Systolic blood pressure; SI, Shock index.

**Table A10:** Patient characteristics in the study population of trauma patients with isolated severe TBI.

	Total (*n* = 831)	Female (*n* = 297)	Male (*n* = 534)	*p*-Value
Age				
Age (years) median (IQR)	62 (16–98)	68 (16–98)	58 (16–95)	<0.001
Age 16–44 years	212 (25.5%)	40 (13.5%)	172 (32.2%)	<0.001
Age ≥ 45 years	619 (74.5%)	257 (86.5%)	362 (67.8%)	<0.001
Injury and severity				
Blunt	808 (98.1%)	294 (99.7%)	514 (97.2%)	0.013
Penetrating	16 (1.9%)	1 (0.3%)	15 (2.8%)	0.013
ISS median (IQR)	25 (16–75)	25 (16–75)	25 (16–75)	0.807
Vital signs				
RTS median (IQR)	6.90 (0–7.84)	6.07 (0–7.84)	6.90 (1.76–7.84)	0.986
RTS < 4	34 (4.1%)	11 (3.7%)	23 (4.3%)	0.674
SBP median (IQR)	141 (45–245)	140 (45–245)	142 (50–240)	0.731
SBP < 90 mmHg	31 (3.7%)	8 (2.7%)	23 (4.3%)	0.239
SI median (IQR)	0.6 (0.2–2.4)	0.6 (0.2–1.6)	0.6 (0.2–2.4)	0.386
Neurological				
GCS median (IQR)	9 (3–15)	8 (3–15)	9 (3–15)	0.883
GCS ≤ 8	370 (44.5%)	139 (46.8%)	231 (43.3%)	0.325
Craniotomy	154 (18.5%)	58 (19.5%)	96 (18.0%)	0.581
ICP measurement	74 (8.9%)	22 (7.4%)	52 (9.7%)	0.258
Comorbidity				
Healthy or mild (ASA ≤ II)	526 (71.1%)	191 (71.0%)	335 (71.1%)	0.972
Severe comorbidity (ASA ≥ III)	214 (28.9%)	78 (29.0%)	136 (28.9%)	0.972
Prehospital				
Prehospital intubation	314 (37.9%)	106 (35.7%)	208 (39.2%)	0.322
P-HEMS	234 (28.2%)	74 (24.9%)	160 (30.0%)	0.121

ASA, American Society of Anesthesiologists; GCS, Glasgow Coma Scale; ICP, Intracranial pressure; IQR, Interquartile range; ISS, Injury Severity Score; P-HEMS, Physician-staffed helicopter emergency medical services; RTS, Revised Trauma Score; SBP, Systolic blood pressure; SI, Shock index.

**Table A11:** Clinical outcomes of trauma patients with isolated severe TBI stratified for sex.

	Total (*n* = 831)	Female (*n* = 297)	Male (*n* = 534)	*p*-Value
Primary outcomes				
In-hospital mortality	234 (28.2%)	94 (31.6%)	140 (26.2%)	0.095
GOS ≤ 3 at discharge	434 (54.3%)	162 (56.8%)	272 (52.9%)	0.286
ICU admission	557 (67.0%)	193 (65.0%)	364 (68.2%)	0.350
Secondary outcomes				
30-day mortality	253 (30.4%)	100 (33.7%)	153 (28.7%)	0.132
ICU days median (IQR)	2 (0–53)	2 (0–39)	2 (0–53)	0.348
ICU > 7 days	201 (24.2%)	65 (21.9%)	136 (25.5%)	0.248
Hospital days median (IQR)	5 (0–135)	5 (0–98)	6 (0–135)	0.074
Hospital > 7 days	376 (45.2%)	126 (42.4%)	250 (46.8%)	0.223
MV admission	424 (51.0%)	145 (48.8%)	279 (52.2%)	0.344
MV days median (IQR)	1 (0–42)	1 (0–33)	1 (0–42)	0.232
MV > 7 days	156 (18.8%)	57 (19.2%)	99 (18.5%)	0.817

GOS, Glasgow Outcome Score; ICU, Intensive Care Unit; IQR, Interquartile range; MV, Mechanical ventilation.

**Table A12:** The association of female sex with clinical outcome parameters in trauma patients presenting with isolated severe TBI.

	OR	95% CI	*p*-Value
Unadjusted values			
In-hospital mortality	1.303	0.954–1.779	0.096
GOS ≤ 3 at discharge	0.853	0.640–1.136	0.277
ICU admission	0.867	0.642–1.170	0.350
30-day mortality	1.264	0.932–1.715	0.132
ICU > 7 days	0.820	0.585–1.148	0.248
Hospital > 7 days	0.837	0.629–1.114	0.223
MV admission	0.872	0.656–1.158	0.344
MV > 7 days	1.044	0.727–1.499	0.817
Adjusted values *			
In-hospital mortality	1.056	0.719–1.550	0.781
GOS ≤ 3 at discharge	1.146	0.794–1.654	0.468
ICU admission	0.909	0.629–1.312	0.609
30-day mortality	0.991	0.674–1.457	0.963
ICU > 7 days	0.786	0.522–1.183	0.248
Hospital > 7 days	0.847	0.621–1.155	0.293
MV admission	0.744	0.481–1.150	0.183
MV > 7 days	1.120	0.714–1.756	0.622

*** corrected for the a priori-determined covariates age, MOI, ISS, RTS < 4, GCS ≤ 8, SI, ASA ≥ III, ICP, and craniotomy. ASA, American Society of Anesthesiologists; GCS, Glasgow Coma Scale; GOS, Glasgow Outcome Score; ICP, Intracranial pressure; ICU, Intensive Care Unit; ISS, Injury Severity Score; MOI, Mechanism of Injury; MV, Mechanical ventilation; RTS, Revised Trauma Score; SI, Shock index.

**Table A13:** Patient characteristics in the study population of trauma patients with isolated severe TBI stratified for age 16–44 years and ≥45 years.

	16–44 Years			≥45 Years		
	Female (*n* = 40)	Male (*n* = 172)	*p*-Value	Female (*n* = 257)	Male (*n* = 362)	*p*-Value
Age						
Age (years) median (IQR)	25 (16–44)	30 (16–44)	0.014	71 (45–98)	68 (45–95)	0.024
Injury and severity						
Blunt	39 (97.5%)	169 (98.3%)	0.570	255 (99.2%)	345 (96.6%)	0.003
Penetrating	1 (2.5%)	3 (1.7%)	0.570	2 (0.8%)	12 (3.3%)	0.003
ISS median (IQR)	23 (16–75)	24 (16–75)	0.202	25 (16–33)	25 (16–75)	0.826
Vital signs						
RTS median (IQR)	5.97 (4.09–7.84)	6.90 (2.63–7.84)	0.612	6.75 (0–7.84)	6.90 (1.76–7.84)	0.765
RTS < 4	0 (0%)	8 (4.7%)	0.357	11 (4.3%)	15 (4.1%)	0.934
SBP median (IQR)	125 (99–170)	134 (55–240)	0.005	144 (45–245)	147 (50–224)	0.660
SBP < 90 mmHg	0 (0%)	6 (3.5%)	0.597	8 (3.1%)	17 (4.7%)	0.324
SI median (IQR)	0.6 (0.4–1.1)	0.6 (0.2–1.9)	0.134	0.5 (0.2–1.6)	0.6 (0.2–2.4)	0.284
Neurological						
GCS median (IQR)	7 (3–15)	9 (3–15)	0.569	9 (3–15)	9 (3–15)	0.998
GCS ≤ 8	20 (50.0%)	76 (44.2%)	0.506	119 (46.3%)	155 (42.8%)	0.390
Craniotomy	5 (12.5%)	34 (19.8%)	0.285	53 (20.6%)	62 (17.1%)	0.271
ICP measurement	6 (15.0%)	20 (11.6%)	0.558	16 (6.2%)	32 (8.8%)	0.231
Comorbidity						
Healthy or mild (ASA ≤ II)	31 (93.9%)	140 (93.3%)	0.087	160 (67.8%)	195 (60.7%)	1.000
Severe comorbidity (ASA ≥ III)	2 (6.1%)	10 (6.7%)	0.087	76 (32.2%)	126 (39.3%)	1.000
Prehospital						
Prehospital intubation	15 (37.5%)	67 (39.2%)	0.322	91 (35.4%)	141 (39.2%)	0.342
P-HEMS	11 (27.5%)	51 (29.7%)	0.788	63 (24.5%)	109 (30.1%)	0.126

ASA, American Society of Anesthesiologists; GCS, Glasgow Coma Scale; ICP, Intracranial pressure; IQR, Interquartile range; ISS, Injury Severity Score; P-HEMS, Physician-staffed helicopter emergency medical services; RTS, Revised Trauma Score; SBP, Systolic blood pressure; SI, Shock index.

**Table A14:** Clinical outcomes of trauma patients with isolated severe TBI stratified for age 16–44 years and ≥45 years.

	16–44 Years			≥45 Years		
	Female (*n* = 40)	Male (*n* = 172)	*p*-Value	Female (*n* = 257)	Male (*n* = 362)	*p*-Value
Primary outcomes						
In-hospital mortality	5 (12.5%)	23 (13.4%)	0.883	89 (34.6%)	117 (32.3%)	0.548
GOS ≤ 3 at discharge	15 (37.5%)	62 (36.0%)	0.877	147 (57.2%)	210 (58.0%)	0.852
ICU admission	27 (67.5%)	119 (69.2%)	0.836	166 (64.6%)	245 (67.7%)	0.423
Secondary outcomes						
30-day mortality	5 (12.5%)	23 (13.4%)	0.883	95 (37.0%)	130 (35.9%)	0.788
ICU days median (IQR)	2 (0–39)	3 (0–47)	0.642	2 (0–34)	2 (0–53)	0.523
ICU > 7 days	8 (29.6%)	47 (39.5%)	0.341	57 (34.3%)	89 (36.3%)	0.487
Hospital days median (IQR)	6 (0–98)	6 (0–93)	0.931	5 (0–74)	6 (0–135)	0.090
Hospital > 7 days	18 (45.0%)	78 (45.3%)	0.968	108 (42.0%)	172 (47.5%)	0.176
MV admission	19 (47.5%)	93 (54.1%)	0.453	126 (49.0%)	186 (51.4%)	0.564
MV days median (IQR)	1 (0–28)	1 (0–42)	0.358	1 (0–33)	1 (0–33)	0.499
MV > 7 days	8 (42.1%)	34 (36.6%)	0.973	49 (38.9%)	65 (34.9%)	0.725

GOS, Glasgow Outcome Score; ICU, Intensive Care Unit; IQR, Interquartile range; MV, Mechanical ventilation.

**Table A15:** The association of female sex with clinical outcome parameters in trauma patients presenting with isolated severe TBI stratified for age 16–44 years and ≥45 years.

	16–44 Years			≥45 Years		
	OR	95% CI	*p*-value	OR	95% CI	*p*-value
Unadjusted values						
In-hospital mortality	0.925	0.329–2.605	0.883	1.109	0.791–1.556	0.548
GOS ≤ 3 at discharge	0.945	0.461–1.936	0.877	1.032	0.740–1.441	0.852
ICU admission	0.925	0.443–1.932	0.836	0.871	0.622–1.221	0.423
30-day mortality	0.925	0.329–2.605	0.883	1.047	0.751–1.459	0.788
ICU > 7 days	0.665	0.286–1.547	0.343	0.874	0.598–1.277	0.487
Hospital > 7 days	0.986	0.494–1.969	0.968	0.801	0.580–1.105	0.177
MV admission	0.769	0.386–1.531	0.454	0.910	0.661–1.253	0.564
MV > 7 days	1.015	0.429–2.400	0.973	1.076	0.714–1.624	0.725
Adjusted values *						
In-hospital mortality	1.083	0.273–4.302	0.910	1.067	0.712–1.600	0.753
GOS ≤ 3 at discharge	0.992	0.375–2.625	0.987	1.241	0.814–1.894	0.316
ICU admission	0.725	0.280–1.875	0.507	0.886	0.585–1.343	0.569
30-day mortality	1.083	0.273–4.302	0.910	0.992	0.660–1.489	0.968
ICU > 7 days	0.378	0.110–1.301	0.123	0.819	0.520–1.289	0.387
Hospital > 7 days	0.907	0.397–2.073	0.818	0.811	0.575–1.142	0.230
MV admission	0.356	0.103–1.226	0.102	0.792	0.493–1.272	0.792
MV > 7 days	1.007	0.321–3.158	0.991	1.087	0.651–1.815	0.750

* corrected for the a priori-determined covariates age, MOI, ISS, RTS < 4, GCS ≤ 8, SI, ASA ≥ III, ICP, and craniotomy. ASA, American Society of Anesthesiologists; GCS, Glasgow Coma Scale; GOS, Glasgow Outcome Score; ICP, Intracranial pressure; ICU, Intensive Care Unit; ISS, Injury Severity Score; MOI, Mechanism of Injury; MV, Mechanical ventilation; RTS, Revised Trauma Score; SI, Shock index.
